# *GENFIT*: software for the analysis of small-angle X-ray and neutron scattering data of macro­molecules in solution

**DOI:** 10.1107/S1600576714005147

**Published:** 2014-05-10

**Authors:** Francesco Spinozzi, Claudio Ferrero, Maria Grazia Ortore, Alejandro De Maria Antolinos, Paolo Mariani

**Affiliations:** a Department DiSVA, Marche Polytechnic University and CNISM, Via Brecce Bianche, I-60131 Ancona, Italy; b European Synchrotron Radiation Facility, Grenoble, France

**Keywords:** *GENFIT*, small-angle X-ray scattering, small-angle neutron scattering, macromolecules

## Abstract

Many research topics in the fields of condensed matter and the life sciences are based on small-angle X-ray and neutron scattering techniques. With the current rapid progress in source brilliance and detector technology, high data fluxes of ever-increasing quality are produced. In order to exploit such a huge quantity of data and richness of information, wider and more sophisticated approaches to data analysis are needed. Presented here is *GENFIT*, a new software tool able to fit small-angle scattering data of randomly oriented macromolecular or nanosized systems according to a wide list of models, including form and structure factors. Batches of curves can be analysed simultaneously in terms of common fitting parameters or by expressing the model parameters *via* physical or phenomenological link functions. The models can also be combined, enabling the user to describe complex heterogeneous systems.

## Abstract 2

*GENFIT* is a new computer code featuring an advanced model-fitting capability to analyse small-angle X-ray and neutron scattering data of macromolecular systems. Batches of experimental curves can be simultaneously best fitted using common parameters issued from combinations of models and, if applicable, constrained by physical and/or phenomenological relations.

## 1. Introduction   

Data collection rates during experiments performed at neutron and, especially, synchrotron sources have increased dramatically in the past few years owing to, among other reasons, ever-increasing source brilliancies and rapid advances in detector technologies. As a result, beamlines now deliver very high flow rates of scientific data and analysts are faced with the challenge of developing software able to cope with the otherwise unavoidable productivity bottlenecks. This also holds for small-angle scattering (SAS) measurements and, in particular, time-resolved or mapping experiments.

Significant progress has recently been made towards a fully automated pipeline encompassing acquisition, reduction and preliminary analysis of small-angle X-ray scattering (SAXS) data, as reported by Franke *et al.* (2012 ▶). For model fitting and in-depth analysis, a large range of software packages designed to analyse both SAXS and small-angle neutron scattering (SANS) data are available to the scientific community at present. A non-exhaustive list of them can be found at the SAS Portal (http://smallangle.org), where the respective application areas are identified. Among the main references in the area of SAS data from biological macromolecules there is *ATSAS*, which is a very extensive and sophisticated set of programs offering the user a rich choice of different shape determination methods as well as various modelling capabilities (Petoukhov *et al.*, 2012 ▶; Graewert & Svergun, 2013 ▶). Besides a number of programs that have been designed for specific aims, there are also multi-purpose program tools, which in general encompass a wide list of models in direct space that can be applied to analyse SAS curves. These programs, which can be included in the so-called ‘direct modelling’ class, are of general interest, in particular for users studying complex systems, such as mixtures of different kinds of particles with or without interaction effects. A list of the most widespread programs of this class, together with their main features, is given in Table 1 ▶.

It is clear that the ever-increasing quality of X-ray and neutron SAS data, together with the dramatic decrease in acquisition time, leads scientists to investigate more and more complex systems and explore to the utmost difficult time-resolved experiments. As a result, scientists are strongly encouraged to design new software tools able to cope simultaneously with many scattering curves and many models, with the aim of deriving not only structural parameters but also ensemble parameters, such as thermodynamic or kinetic functions. In the light of this and of the user’s quest for accurate and reliable modelling abilities, we have developed the program *GENFIT*, targeting the following list of requirements:

(*a*) Fitting large experimental data sets by the selection of one or more models that can be suitably combined from a repository of over 30 models, ranging from simple asymptotic behaviours (*e.g.* Guinier and Porod laws) up to complex geometric architectures or entirely atomic structures.

(*b*) Providing form- and structure-factor based models that take into account interactions between particles in solution.

(*c*) Supplying a model-fitting approach which intrinsically allows for polydisperse distributions of particles of arbitrary form having an internal structure.

(*d*) Featuring the ability to relate the parameters of the theoretical models to experimental chemical–physical conditions (temperature, pressure, concentration, pH, ionic strength *etc.*), *e.g.* by means of user-defined link-functions.

(*e*) Generating theoretical SAS curves based on model assumptions or on knowledge of the species in solution, with the aim of predicting the optimum experimental conditions to be explored in a prospective SAS experiment.

(*f*) Offering an open-source distribution mechanism which enables end users to contribute their own models to the *GENFIT* scope *via* a simple plug-in architecture. Today, more than ever, the visibility and testability of the internal structure of a software package is required by the scientific community in a common effort towards transparency of process with the public bodies representing tax payers across different countries.

## 2. Features of *GENFIT*   

*GENFIT* is written in Fortran and a simple-to-use and modular graphical user interface (GUI) has been added. The *GENFIT* GUI has been designed so as to evolve at the same pace as the related code and to enable the efficient use of the program, even online during a campaign of measurements with generally little time availability.

In the following sections we provide an overview of the main features of *GENFIT*, making use of sample data recorded mainly at European large-scale facilities.

### 2.1. Input SAS curves and the *GENFIT* GUI   

The input data for *GENFIT* are experimental one-dimensional SAS curves, usually taken to be the macroscopic differential scattering cross section, indicated here as *I*_exp_(*q*), as a function of the modulus of the momentum transfer, *q* = (4π/λ)sinθ, where θ is half the scattering angle and λ is the wavelength of the incident radiation. If the SAS experiment has been correctly calibrated, *I*_exp_(*q*) is given in absolute units, usually cm^−1^. However, data in arbitrary units are also treated by *GENFIT*. An experimental SAS curve is normally written in a three-column ASCII file, with *q*, *I*_exp_(*q*) and its standard deviation σ(*q*) in the first, second and third column, respectively. Numbers can be expressed in any format. If standard deviations are not provided in the data file, they can be generated using a simple power-law expression, σ(*q*) = *k*[*I*_exp_(*q*)]^α^.

**Figure 1:**
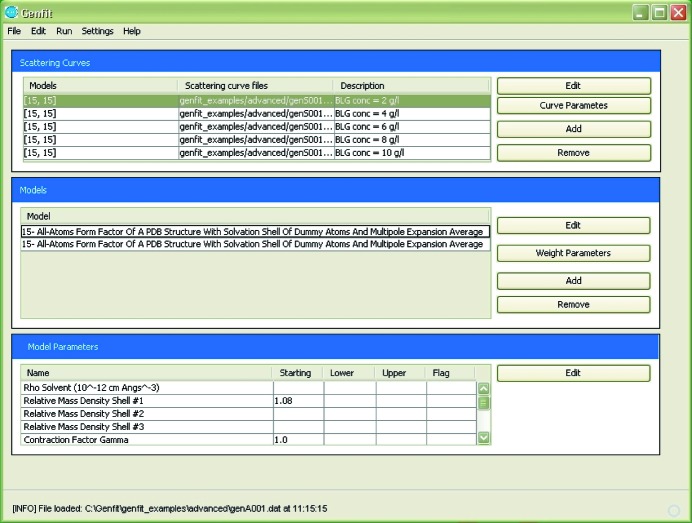
The main window of the *GENFIT* GUI. The top, middle and bottom sections display information on the scattering curves, the models applied to analyse the scattering curves and their respective parameters. Detailed information regarding each section is supplied by the user by activating the buttons on the right-hand side. Commands in the menu bar allow opening a *GENFIT* (File) input file, selecting the χ^2^ minimization methods (Edit), executing the calculation and exploring the results (Run), and managing the settings parameters of the software (Settings).

The GUI of *GENFIT* assists the user in loading experimental curves, selecting models, executing the fitting calculation, viewing the output files and showing the fitting curves using *GNUPLOT* (Williams *et al.*, 2010 ▶). The GUI is written in Java and comprises three main sections, as displayed in Fig. 1 ▶.

Smearing effects are taken into account using the procedure described by Pedersen *et al.* (1990 ▶), where each effect contributes to the width of a Gaussian curve, which is then used in a convolution integral applied to the model scattering intensity. The convolution integral is actually computed using the flag Collimation. Vertical and horizontal slit effects are also accounted for in the calculation, as described by Glatter & Kratky (1982 ▶).

### 2.2. Global fit   

One of the distinctive features of *GENFIT* is the ability to analyse more than one experimental SAS curve at a time, a way of proceeding indicated by the term ‘global fit’. This task is accomplished by minimizing the standard reduced χ^2^ function, defined for a set of *N*_*c*_ experimental SAS curves *I*_exp,*c*_(*q*) as
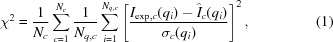
where *N*_*q*,*c*_ is the number of *q* points on curve *c* and 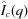 is the fitted SAS curve as determined by *GENFIT*. In order to make allowance for data in arbitrary units and/or the possible presence of a flat scattering signal (for example the incoherent background of a neutron scattering experiment), the fitted SAS curve is written as 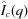 = κ_*c*_ *I*_*c*_(*q*) + *B*_*c*_, where *I*_*c*_(*q*) is the model SAS curve expressed in absolute units. The scaling factor κ_*c*_ and the background *B*_*c*_ can be fixed by the user or are easily calculated using standard linear least-squares minimization (Press *et al.*, 1994 ▶).

### 2.3. Model scattering curve   

The general object of *GENFIT* is to depict the SAS curve, *I*_*c*_(*q*), intended to fit the experimental curve *c*, as a linear combination of *M*_*c*_ models:
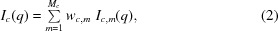
where *w*_*c*,*m*_ is the weight of the *m*th model curve, *I*_*c*,*m*_(*q*), that contributes to the best fit. This model depends typically on a set of *P*_*m*_ unknown parameters, here indicated as *X*_*c*,*m*,1_, *X*_*c*,*m*,2_, …, *X*_*c*,*m*,*P*_*m*__ and called ‘model parameters’. They are, in general, structural parameters, such as thickness, scattering length density, electric charge and so on. Each model parameter can be associated with a flag which determines whether the parameter is fixed or fitted. Moreover, the flag indicates whether the model parameter is linked to one or more experimental SAS curves, or is rather involved in a physical or phenomenological function. The various flag utilities are described in §§2.6–2.8 [Sec sec2.8]. Weights and model parameters are estimated by minimizing the χ^2^ distribution [equation (1)]. The GUI assists the user in associating with each of the experimental curves the *M*_*c*_ models, which can be selected from a list including more than 30 items and which is continuously upgraded. Notice that in equation (2) the index *m* is a counter for the number of models used to analyse curve *c*. This number is different from the number μ that *GENFIT* uses to label a model within the list of all the models that the program can handle (see §S1 in the supporting information^1^).

### 2.4. PDB-based models   

Several models included in *GENFIT* are able to calculate the form factors of atomic structures on the basis of Protein Data Bank (PDB) files (Berman *et al.*, 2000 ▶), taking into account the contribution of the solvation shell around the macromolecule. Some models make use of a Monte Carlo approach (Mariani *et al.*, 2000 ▶; Spinozzi *et al.*, 2000 ▶, 2002 ▶), whereas others are based on the recently developed *SASMOL* method (Ortore *et al.*, 2009 ▶, 2011 ▶), which uses the spherical harmonic expansion of the scattering amplitudes, similar to the widely known *CRYSOL* software (Svergun *et al.*, 1995 ▶). The main idea of *SASMOL* is to embed the macromolecule in a ‘tetrahedrical close-packed’ lattice and assign the lattice positions in contact with the atoms of the macromolecule to hydration molecules. In this way, the scattering contribution of water molecules inside cavities or grooves is taken into account. For each of the PDB-based models, the GUI provides a facility where the user can load the PDB files.

### 2.5. Structure factors   

Some of the models included in *GENFIT* are defined in terms of both form factor, *P*(*q*), and structure factor, *S*(*q*). The latter is calculated within the framework of the most popular approximations for monodisperse systems, such as the mean spherical approximation (Hayter & Penfold, 1981 ▶; Hansen & Hayter, 1982 ▶) and the random phase approximation (Narayanan & Liu, 2003 ▶; Barbosa *et al.*, 2010 ▶). For systems composed of a mixture of oligomeric species, the first-order approximation of the expansion of the mean force potential into a power series of the overall monomer number density is used (Spinozzi *et al.*, 2002 ▶; Gazzillo *et al.*, 2008 ▶). Cluster structures of particles with different shapes are described by the structure factor developed by Teixeira (1988 ▶). One- or two-dimensional correlations among lipid bilayers dispersed in water are analysed *via* the paracrystal theory (Hosemann & Bagchi, 1952 ▶; Matsuoka *et al.*, 1987 ▶; Frühwirth *et al.*, 2004 ▶) or the modified Caillé theory (MCT) (Zhang *et al.*, 1994 ▶, 1996 ▶).

### 2.6. Basic calculation of parameters   

**Figure 2:**
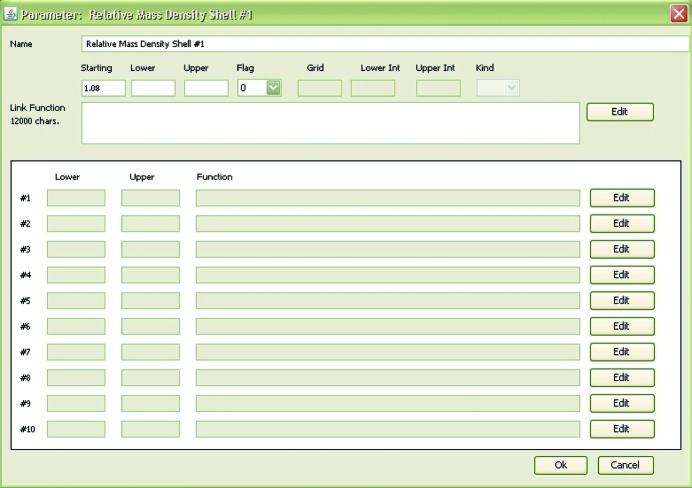
The GUI parameter window, showing the name of the parameter (top field), its Starting, Lower and Upper values (second row, left), and the possible link function (third row, left). Through the Flag field the user can control the way *GENFIT* should handle the parameter, as described in the text. In the case of polydispersity, the setting values for the integration [equation (3)] are entered using the fields in the second row on the right. Lower and Upper values of the parameters defining the polydispersity model, together with their possible link functions, are managed in the last ten rows of the window.

*GENFIT* prompts the user to specify how to handle both the weights, *w*_*c*,*m*_, and the model parameters, *X*_*c*,*m*,*k*_. The way this is done in *GENFIT* is by setting a starting value of a parameter together with its lower and upper values, hence three fields, called Starting, Lower and Upper, are correspondingly filled (Fig. 2 ▶). It may be that some of the parameters are known from *a priori* information on the system. In order to make provision for such cases, each parameter within *GENFIT* is associated with a Flag: if Flag = 0 the parameter is considered fixed to the value indicated in the Starting field, whereas if Flag = 1 the parameter is optimized in the range between Lower and Upper values. If the same model μ is used to fit more than one curve within the set of *N*_c_ SAS curves, some of its parameters can be defined by the user as ‘common parameters’, the values of which should be shared by all the curves *I*_*c*,*m*_(*q*) adopting model μ. This information can be passed on to *GENFIT* by associating the value Flag = 2 with all the common parameters (*w*_*c*,*m*_ or *X*_*c*,*m*,*k*_).

### 2.7. Polydispersity   

In several circumstances the model parameters *X*_*c*,*m*,*k*_ can be distributed over a range of values, represented by a polydispersity function. When the *k* parameter is polydisperse, the average scattering curve of model *m* is written as an integral over the distribution function *f*_*c*,*m*,*k*_(*X*_*c*,*m*,*k*_):
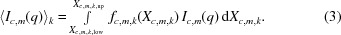
This equation can be generalized to the case of more than one polydisperse parameter. Assuming, for the sake of simplicity, that the unique polydispersity distribution function *f*(*X*_*c*,*m*,1_, *X*_*c*,*m*,2_, …, *X*_*c*,*m*,*N*_) can be expressed as the product of the distribution functions related to each parameter *X*_*c*,*m*,*k*_ (decoupling approximation), then equation (3) can be repeatedly applied to all the polydisperse parameters:
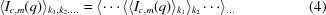
However, the decoupling approximation cannot be applied to all investigated systems: the user should be aware of this fact and, just in case, examine the results critically.

By selecting Flag = 6 in association with the parameter *X*_*c*,*m*,*k*_, *GENFIT* builds a polydispersity function over this parameter (Fig. 2 ▶). In the most recent version of the program, seven different kinds of polydispersity model have been implemented (see §S2 in the supporting information). Each polydispersity model includes some parameters that *GENFIT* is expected to optimize. If the polydispersity parameters related to *X*_*c*,*m*,*k*_ are considered ‘common parameters’, shared by all the curves *I*_*c*,*m*_(*q*) adopting model μ, the corresponding flag should be fixed to Flag = 7.

### 2.8. Calculation of parameters through link functions   

The user might see good reasons to apply some constraints to the weights or model parameters. As an example, in the case of a mixture of different oligomers, the weights of the models describing each oligomer should be linked to the nominal concentration of the sample, which the user probably knows. Another example could be the case of curves recorded at different temperatures: the user could try to check whether the fitting parameters are linear or exponential functions of temperature. On the other hand, one would possibly like to combine structural models able to fit the SAS curves with chemical–physical models suitable for describing, for example, the dependence of some species on concentration, temperature, pressure and so on. In order to encompass such complex and interesting cases, *GENFIT* allows the user to define a parameter (*w*_*c*,*m*_ or *X*_*c*,*m*,*k*_) through a ‘link function’. This option is activated by entering Flag = 4 and writing in the field named Link Function the expression that *GENFIT* will use to calculate the parameter. In general, expressions are written as functions of coefficients that are classified into two groups within *GENFIT*. Coefficients that characterize each experimental SAS curve (such as temperature, pressure, concentration *etc.*) are referred to as ‘*p*-coefficients’ and are not adjustable. All other coefficients can in principle be adjusted and are called ‘*f*-coefficients’. A link function can contain both *p*- and *f*-coefficients. For instance, if the user has defined among the *p*-coefficients the temperature as temp and wishes to impose linear behaviour on a model parameter *X*_*c*,*m*,*k*_ *versus* temperature, the Link Function associated with *X*_*c*,*m*,*k*_ can be written as a+b*temp. *GENFIT* recognizes that a and b are *f*-coefficients associated with the *c* curve to be fitted. Through Flag = 5 a more general case can be introduced: all the *f*-coefficients (a and b in the example above) that *GENFIT* finds in the link function are considered ‘common parameters’ of the set of *N*_*c*_ curves.

The parameters of the polydispersity models introduced in §2.7 can also be expressed using link functions, which can include either *p*- or *f*-coefficients or both. The polydispersity option is selected either by Flag = 8, indicating that all the *f*-coefficients that appear in the link function pertain to curve *c*, or by Flag = 9, allowing the whole set of *f*-coefficients to be common to all the *N*_*c*_ SAS curves.

### 2.9. File of parameters   

All parameters optimized by *GENFIT* in a run are reported at the end of the calculation in a ‘file of parameters’, which is named gen<code>.par, where <code> is a four-character alphanumeric label assigned to the calculation. Each row in the file refers to a parameter and is made up of six figures: the ordinal number of the parameter, its name, its final value, its standard deviation, and its lower and upper limits. If the parameter is a basic parameter of a model (*w*_*c*,*m*_ or *X*_*c*,*m*,*k*_), the upper and lower limits are the values indicated by the user in the respective menu (see Fig. 2 ▶). When at least one of the adjustable parameters is an *f*-coefficient (a situation that occurs when the user has written at least one link function to calculate a parameter), the first execution of *GENFIT* is aimed not at minimizing χ^2^ but only at generating a file of parameters gen<code>.par, where the upper and lower limits of the *f*-coefficients are set by default to 0 and 1, respectively. The user can modify the default limits of the *f*-coefficients by editing the file gen<code>.par. In the second run, *GENFIT* will read the modified gen<code>.par file and execute the χ^2^ minimization using the new lower and upper limits for the *f*-coefficients.

### 2.10. Penalty function   

An estimation process in which the likelihood is augmented by a function of the fitting parameters is often desirable, depending on the physical meaning of the parameters, even though the goodness of the fit, as determined by the χ^2^ function [equation (1)], is not modified. Hence, *GENFIT* allows the user freely to define a ‘penalty function’ Ψ which will be added to χ^2^. The variable name reserved for the penalty function Ψ is fout. The value of fout is set to zero before starting the calculation of the fitting parameters. The user can define the value of fout within a link function. At the end of the minimization the value of Ψ is reported in the output file of *GENFIT*, together with χ^2^ (see below). The user can judge whether Ψ is too high or too low with respect to χ^2^ and change the definition of fout accordingly.

### 2.11. Minimization of χ^2^   

The minimization of χ^2^ [equation (1)], with the possible addition of the penalty function Ψ (see §2.10), can be performed by selecting from four different methods: (i) monkey, (ii) simulated annealing, (iii) simplex and (iv) quasi-Newton. Details are reported in §S3 of the supporting information. The Hessian matrix calculated by the quasi-Newton method is also used to estimate the uncertainty in the fitting parameters and their correlation matrix. A more robust calculation of the parameter errors can be obtained by iteratively moving all the points of the experimental SAS curves within their standard deviations, by repeating the minimization and calculating the mean value and standard deviation of each fitting parameter after *N*_*I*_ iterations.

### 2.12. Output files   

At the end of the calculation, *GENFIT* generates a number of output files which include, among others, best fitting curves, parameters, distribution functions of the polydisperse parameters and Fourier transforms. The name and scope of each output file are reported in §S4 of the supporting information.

## 3. Examples   

In order to illustrate the main *GENFIT* features, a few examples of SAS data analysis are reported in the following sections. It should be noted that the cases discussed refer to experiments performed at synchrotron beamlines or using simulated data.

### 3.1. Oligomeric association   

It is well known that, under physiological conditions, biological macromolecules can be found at relatively high concentrations and also, as observed in several biologically relevant cases, in different aggregation states (Baldini *et al.*, 1999 ▶; Barbosa *et al.*, 2010 ▶; Spinozzi *et al.*, 2012 ▶). SAS experiments performed on concentrated solutions can be very useful to derive information on the different species present at equilibrium, including aggregation number and concentration. However, the data analysis can be very difficult, although if simple internal constraints are used a good deal of information can be extracted. Indeed, in the case of negligible interactions between particles in solution, the macroscopic differential scattering cross section *I*(*q*) can be written as the sum of the weighted contributions of the form factors for the different oligomeric states: because the macromolecular concentration of the solution is known and because the thermodynamics of the aggregating species can be described in terms of dissociation constants, the weight parameters for each form factor should correlate with the dissociation free energies and the experimental conditions of the sample, such as molar concentration, pressure and/or temperature (Baldini *et al.*, 1999 ▶; Spinozzi *et al.*, 2003 ▶; Ortore *et al.*, 2005 ▶). Using *GENFIT*, such relations may be transformed to link functions that can be used during the SAS curve-fitting procedures to converge to a stable and well defined result.

As the understanding of protein aggregation is a central issue in different fields, from heterologous protein production in biotechnology to amyloid aggregation in many neurodegenerative and systemic diseases, we focus on an example concerning protein oligomerization and present the case of β-lactoglobulin (BLG), an 18 400 Da protein belonging to the lipocaline family. This protein can be found in solution in both monomeric and dimeric states and it is known that the association behaviour can be influenced by protein concentration, ionic strength (Schaink & Smit, 2000 ▶; Baldini *et al.*, 1999 ▶; Spinozzi *et al.*, 2002 ▶), temperature and pressure (Valente-Mesquita *et al.*, 1998 ▶; Ortore *et al.*, 2005 ▶).

**Figure 3:**
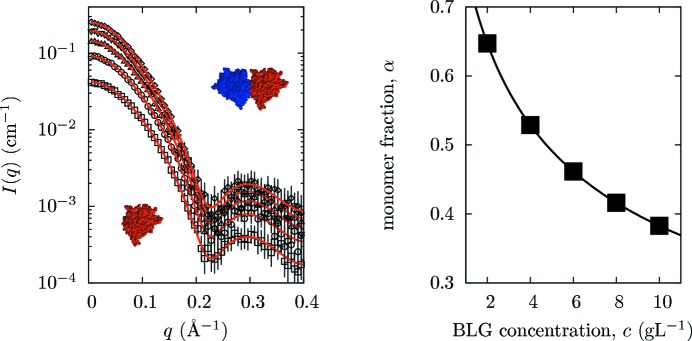
(Left) SAXS simulated curves obtained at increasing BLG concentration in solution (from bottom to top, open squares, circles, up-triangles, down-triangles and diamonds correspond to 2, 4, 6, 8 and 10 g l^−1^, respectively) and their best fits obtained with *GENFIT* (solid red lines). All SAXS data were simulated at ambient pressure and temperature, at pH 2.3, and at 100 m*M* ionic strength. The structures of the BLG monomer and dimer are depicted using the *Rasmol* software (Bernstein *et al.*, 2000 ▶). The best fit values of the dissociation free energy and the relative mass density of the hydration shell are Δ*G*_dis_/(*k*_B_ *T*) = 8.22 ± 0.08 and 1.08 ± 0.01, respectively. (Right) BLG monomer fraction in solution *versus* BLG concentration as obtained from the dissociation free energy.

This BLG example shows how *GENFIT* can be exploited to derive thermodynamic parameters from a batch of SAS curves. To this end, a number of SAXS curves were generated for increasing BLG concentrations from 2 to 10 g l^−1^. As the BLG dissociation free energy at ambient pressure and temperature, pH 2.3 and an ionic strength of 100 m*M* is known (Δ*G*_dis_ = 8 *k*_B_ *T*, *k*_B_ being the Boltzmann constant and *T* the temperature; Baldini *et al.*, 1999 ▶), SAXS curves were simulated considering the actual fraction of monomers and dimers of BLG in solution and their form factors, as derived by applying to the corresponding PDB coordinate files the spherical harmonics approach of the *SASMOL* tool, described in §2.4 and implemented in the *GENFIT* suite. Since experimental curves were simulated at rather low BLG concentrations (≤1% *w*/*w*), protein–protein interactions were neglected and the structure factor *S*(*q*) approximated to unity. Simulated curves are shown in Fig. 3 ▶. Note that, to approximate a real experiment, any point on the calculated curves has been randomly moved by sampling from a Gaussian distribution with mean *I*_*c*_(*q*) and standard deviation σ(*q*) = *k*[*I*_*c*_(*q*)]^1/2^. The constant *k* was chosen in order to obtain a relative error of 3% for the first point of the simulated curve.

After the numerical simulations, the *GENFIT* global fitting procedure was applied to all the curves using BLG dimer and monomer structures obtained from the PDB and keeping as common fitting parameters the dissociation free energy Δ*G*_dis_ and the relative mass density of the protein hydration shell. In particular, the following link functions were used to connect the form factor weight parameters *w*_mon_ (for the monomer) and *w*_dim_ (for the dimer) to the nominal protein weight concentration *C* and experimental temperature *T*:
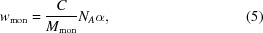


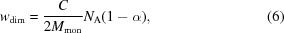
where *N*_A_ is Avogadro’s number, *M*_mon_ is the monomer molecular weight and α is the fraction of monomers in solution,
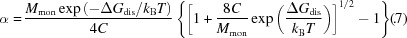
Note that the dissociation constant is in fact 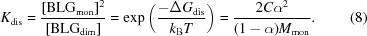
Best fitting curves are shown in Fig. 3 ▶, where it can be observed that the global fitting procedure reproduces the simulated curves well. Moreover, the resulting common fitting parameters, Δ*G*_dis_ and the relative mass density of the protein hydration shell, appear very consistent with the values used in the numerical simulation.

### 3.2. Unfolding processes   

Protein unfolding is another scientific issue widely investigated by SAXS/SANS techniques. In fact, even the radius of gyration obtained by Guinier analysis (Guinier & Fournet, 1955 ▶) of a SAS experimental curve readily provides an initial and meaningful indication of protein compactness, and hence of its folding/unfolding state. However, a deeper analysis of the unfolding process, which proceeds under the control of denaturing agents such as temperature, pressure, pH or concentration of cosolvents, should take into account the equilibrium between folded and unfolded species present in solution. As in the previous case, the application of *GENFIT* link functions and the extended use of common fitting parameters allows the determination of crucial factors.

**Figure 4:**
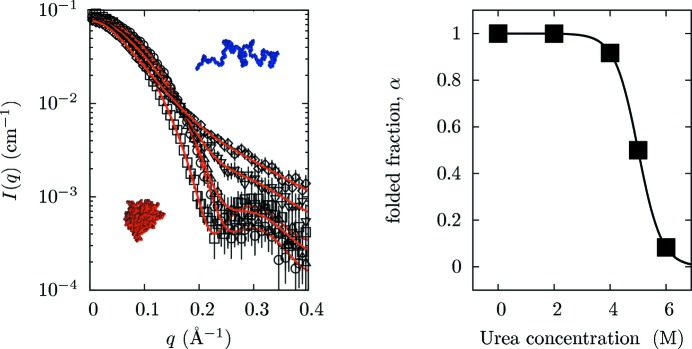
(Left) Simulated SANS curves obtained for BLG in D_2_O at 5 g l^−1^ with increasing urea concentration in solution (from bottom to top, open squares, circles, up-triangles, down-triangles and diamonds correspond to 0, 2, 4, 5 and 6 *M* urea, respectively) and their best fits obtained with *GENFIT*. All data were simulated at ambient pressure and temperature, at pD = 2.3, and at 20 m*M* ionic strength. The native BLG monomer and the unfolded chain are reported. The best fit parameters of the worm-like monomer were Kuhn length *b* = 4.6 ± 0.4 Å, inner cross section *R* = 4.1 ± 0.2 Å, number of statistical segments *N*_b_ = 90 ± 20 and relative mass density of the hydration shell *d*_w_ = 0.951 ± 0.001. The best fit parameters of the unfolding free energy are Δ*G*_unf,0_ = 12 ± 1 *k*_B_ *T*, Δ*G*_unf,1_ = −2.4 ± 0.2 *k*_B_ *T* *M* ^−1^ and Δ*G*_unf,2_ = 0.00 ± 0.03 *k*_B_ *T* *M* ^−2^. (Right) BLG folded monomer fraction in solution *versus* urea molar content as obtained from the calculated unfolding free energy.

In this example, we simulated a set of SANS curves for BLG dissolved in D_2_O at a fixed concentration but with an increasing content of urea (see Fig. 4 ▶). The SANS contribution of BLG monomers in their native conformation was simulated according to the form factor derived from PDB entry 1beb (Brownlow *et al.*, 1997 ▶), while the contribution from unfolded monomers was obtained using a worm-like model with excluded volume, described originally by Pedersen & Schurtenberger (1996 ▶) (the fixed parameters of the worm-like model were Kuhn length *b* = 4.2 Å, inner cross section *R* = 4.0 Å, number of statistical segments *N*_b_ = 100, and thickness and relative mass density of the hydration shell δ = 3 Å and *d*_w_ = 0.95, respectively). The relative fraction of native and unfolded BLG particles in solution was established to depend on the urea molar concentration [U]. Therefore, considering the folding–unfolding equilibrium, the concentration of the two species was calculated using an unfolding free energy defined by 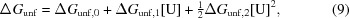
with Δ*G*_unf,0_ = 10.5 *k*_B_ *T*, Δ*G*_unf,1_ = −2.06 *k*_B_ *T* *M* ^−1^ and Δ*G*_unf,2_ = −0.0026 *k*_B_ *T* *M* ^−2^. The five SANS curves in D_2_O, simulated at different values of [U] and altered to include experimental errors, are shown in Fig. 4 ▶.

SANS data were fitted globally with *GENFIT*, using a link function to bind the unfolding free energy, nominal protein concentration, urea concentration and form-factor weight parameters, and optimizing all common parameters describing the unfolding free-energy dependence on [U] and the unfolded BLG. As in the previous example, it can be seen from Fig. 4 ▶ that the *GENFIT* results reproduce the simulated data quite well, yielding fitting parameters (shown in the figure caption) very close to those used in the simulations.

### 3.3. Multilamellar vesicles   

SAS techniques are largely used to provide information on the structural properties of vesicular systems at the nanoscale level. In particular, owing to the importance of some kinds of vesicles in the context of drug delivery, SAXS/SANS can be crucial to elucidate the inner structure of nanoparticles, *i.e.* when the uni- or multilamellar nature of the particles is unknown.

The example of SDS/CTAB cat–anionic vesicles, which present critical temperature behaviour, can be very instructive (Andreozzi *et al.*, 2010 ▶; SDS is sodium dodecylsulfate and CTAB is cetyltri­methyl­ammonium bromide). Cat–anionic vesicles are mixtures of oppositely charged surfactants that exhibit a phase behaviour in water very similar to that occurring in natural lipids, with the formation of micelles, multilamellar and unilamellar vesicles, solids, and lyotropic mesophases. Since cat–anionic mixtures are moderately cytotoxic, they have been used extensively in studies dealing with protein uptake or DNA transfection.

**Figure 5:**
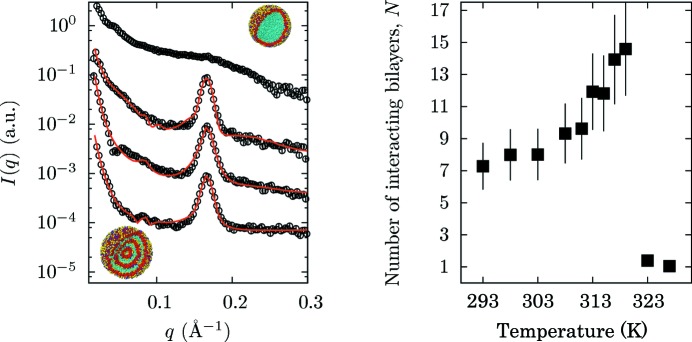
(Left) Experimental SAXS curves referring to vesicles with composition according to the ratio SDS:CTAB = 1.71 and overall surfactant content equal to 6.0 mmol kg^−1^. From the bottom curve to the top the temperature values are 303, 308, 311 and 323 K. For temperatures lower than 323 K, the curve best fits obtained by *GENFIT* as described in §3.3 are also reported. The curves are scaled for the sake of clarity. Multi- and unilamellar vesicle cartoons are featured. (Right) The number *N* of the resulting interacting bilayers as a function of temperature for the whole set of experimental curves (Andreozzi *et al.*, 2010 ▶).

SDS/CTAB cat–anionic vesicles were recently analysed by SAXS at the DESY synchrotron in Hamburg, Germany (Andreozzi *et al.*, 2010 ▶). A few experimental scattering curves are reported in Fig. 5 ▶, and it can be observed that Bragg peaks are present at low temperatures, confirming the multilamellar nature of the vesicles. These peaks disappear on heating, suggesting that increasing the temperature induces a transition to a different vesicle structure, probably uni­lamellar. A global fitting analysis of the whole set of scattering curves was performed using a form factor for the lamella coupled with a structure factor related to the bilayer stacking order. The form factor was described by the Fourier transform of the electron-density distribution normal to the bilayer plane, accounting for water and polar and hydrocarbon regions with smooth interfaces [see Fig. 7 of Andreozzi *et al.* (2010 ▶)], while the structure factor was modelled according to the MCT (see §2.5), both implemented in *GENFIT*.

The final fitting results provide not only basic information on the bilayer structure but also a determination of the number of strongly interacting bilayers, *N*, and of their fluctuation parameter, which is in turn related to the bending modulus *k*_C_ of the bilayer and the bulk compression modulus *B*. In particular, an increase in bilayer thickness on heating and a corresponding decrease in the value of *k*_C_ *B*, which indicates a significant softening of the lamellar stack as a function of temperature, were detected. Moreover, the number of strongly interacting bilayers was observed to increase up to the critical temperature at which the transition to unilamellar vesicles takes place, indicating that vesicle growth and/or fusion occurs before the transition.

This example underlines the benefit of an analysis of SAXS data based on convenient models, so the technique can be regarded as a complementary tool to microscopies and/or dynamic light scattering (DLS). Indeed, in the present case the overall changes in vesicle size established by DLS were discovered to be concomitant with the inner structural changes described here.

### 3.4. Guanosine association   

SAS has also been used to monitor complex aggregation/fragmentation processes in solution (Mariani *et al.*, 2009 ▶, 2010 ▶; Gonnelli *et al.*, 2013 ▶). In particular, the possibility of defining link functions and global parameters in the *GENFIT* data analysis process allowed several guanosine aggregate species formed by self-assembly in solution to be resolved in terms of concentration and composition.

**Figure 6:**
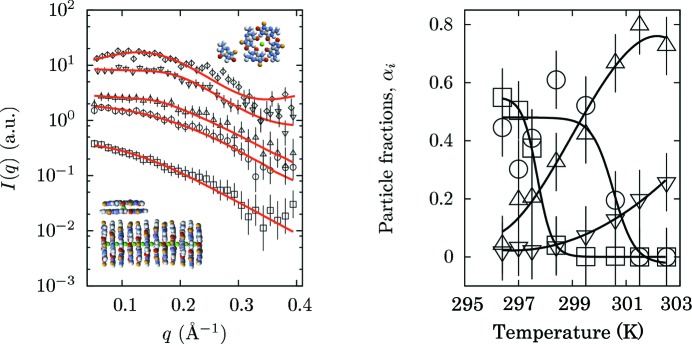
(Left) Experimental SAXS curves referring to d(pG) at 5 wt% concentration and different temperatures. From the bottom to the top: open squares 296.6 K, open circles 297.7 K, up-triangles 298.6 K, down-triangles 300.8 K, diamonds 302.7 K. The solid lines are the *GENFIT* global fit curves. The scattering curves are scaled by an appropriate factor for the sake of clarity. (Right) Temperature dependence of the fraction of particles assembled in different forms. Down-triangles correspond to monomers, up-triangles to quartets, squares to octamers and circles to quadruplexes.

Here we describe the case of the temperature behaviour of 2-deoxyriboguanosine 5′-monophosphate, d(pG), which auto-assembles in aqueous solution in the form of quartets, octamers and pseudo-polymeric quadruplexes characterized by the absence of a covalent axial backbone (Mariani *et al.*, 2009 ▶). As contradictory findings have been reported in the literature, the effect of temperature on d(pG) self-assembly was investigated in particular (Mariani *et al.*, 2009 ▶). Some of the experimental SAXS curves recorded at the ELETTRA synchrotron in Trieste, Italy, are shown in Fig. 6 ▶. A very different behaviour can be readily observed, as the SAXS profiles at low temperature show a strong small-angle intensity, while the curves at higher temperature are characterized by a very diffuse and low-intensity band.

A *GENFIT* global fitting approach was used to derive the concentrations and sizes of the different scattering particles existing in solution, as a function of temperature. In particular, the form factors for d(pG) and G quartets were calculated from PDB atomic structures, while G quadruplexes were represented as monodisperse right circular cylinders with a core–shell electron-density profile. The concentrations of the different particles formed and the length of the quadruplexes were fitted curve by curve, under the constraint of a constant nominal concentration. The radius and shell thickness of the cylindrical model, and the electron densities of the core and shell regions of the cylinder, were considered as global parameters and fitted simultaneously on the entire set of SAXS curves obtained at increasing temperature. In Fig. 6 ▶, best fit curves are superimposed on the experimental SAXS data so that the very good quality of the fitting procedure can be appreciated. The figure also shows the relative composition of the different guanosine aggregates occurring in solution as a function of temperature. The results are very interesting, as it appears that the various d(pG) structures exhibit different thermal stability trends. Octamers are stable up to 298 K, when their fragmentation begins and the number of both free d(pG) molecules and G tetramers increases. On the other hand, the G quadruplexes shorten at higher temperatures and disappear at around 301 K. In summary, two melting processes occur, featuring the two-step mechanism of d(pG) self-assembly.

## 4. Summary, conclusions and outlook   

*GENFIT* is a software package to analyse sets of SAS curves recorded from nanosized macromolecular systems using one or more suitable models, which contain both form and structure factors. The parameters of the models are optimized in a versatile manner, enabling the user to easily impose constraints or to express them through suitable functions. Such functions can be simple phenomenological relationships or chemical–physical laws. This approach is particularly useful when a set of SAS curves has been obtained for the system of interest by varying one or more external conditions. In such cases, the *GENFIT* analysis of the whole set of SAS curves can extract relevant physical information (for example thermodynamic parameters) that describes the behaviour of the system under the investigated conditions. *GENFIT* can be useful for optimizing the steps of a SAS study and for exploiting fully the complementarity between SAXS and SANS. It allows the simulation of SAS curves and testing of whether, by analysing them as single measurements or as a whole set of measurements, it is actually possible to recover the information the user is interested in. A GUI has been developed to assist the user in exploiting all the *GENFIT* characteristics in a simple and intuitive way. *GENFIT* runs under Windows, Linux and MacOS and is freely available from the distribution web site (Spinozzi, 2013 ▶). It is open source for registered users (registration is free of charge). *GENFIT* is modular software, and new models and features are continually integrated into it by the authors.

It should be noted that a set of guidelines for the presentation of SAS results in structural molecular biology has recently been published (Jacques *et al.*, 2012 ▶). Such guidelines would ensure adequate SAS data reporting and analysis, but would also give a warning about the risk of model overparameterization (*i.e.* the introduction of more parameters into the model used to fit the SAS data than can be justified). It is evident that *GENFIT* is not concerned with data reduction or presentation, but the use of *GENFIT* can certainly reduce the risk of overparameterization. In fact, the extended use of link functions, which add restraints based on complementary physical–chemical and/or thermodynamic information, as well as the global fit approach (Ortore *et al.*, 2011 ▶), should help the user in reducing the number of parameters and providing a proper justification for the specific modelling protocol employed.

## Supplementary Material

Supporting information file. DOI: 10.1107/S1600576714005147/to5062sup1.pdf

## Figures and Tables

**Table 1:** Overview of the most widespread programs to analyse SAS data by the direct modelling approach

Program	Features	Global fit
*FISH* (Heenan, 2005 ▶)	A limited number of data sets may be fitted simultaneously to the same model. Size polydispersity and some constraints, such as known molecular volumes or shell thicknesses, may also be incorporated. The models are grouped by functionality, and a structure factor *S*(*q*) multiplies the previously accumulated form factor(s).	Yes
*IRENA* (Ilavsky & Jemian, 2009 ▶)	Package typically deployed for the analysis of SAS data in materials science, chemistry, polymers, metallurgy, and the physics of solid or liquid samples. It addresses complex systems with size distributions, hierarchical structures, diffraction peaks *etc.*	Yes
*NCNR* (Kline, 2006 ▶)	Data reduction and analysis of SANS and USANS data on the basis of model-independent methods or nonlinear fitting deploying a large catalogue of structural models. Smearing effects can be accounted for automatically during analysis and any number of data sets can be analysed simultaneously. Models and data-reduction operations allow users to contribute their code and models for general distribution.	No
*SASfit* (Kohlbrecher & Bressler, 2006 ▶)	The program has been written for analysing and displaying SAS data. It can calculate integral structural parameters like radius of gyration, scattering invariant, Porod constant and so forth. Furthermore, it can fit size distributions together with several form factors, including different structure factors. A global fitting algorithm has been implemented in *SASfit*, which allows the simultaneous fitting of several scattering curves using a common set of parameters. The global fit helps to determine model parameters unambiguously, which could possibly suffer from strong correlation if one analyses only an individual curve.	Yes

## Footnotes

1 Supporting information discussed in this paper is available from the IUCr electronic archives (Reference: TO5062). For additional information on the models and methods used, see Aird (1984 ▶), Beaucage (1996 ▶), Cinelli *et al.* (2001 ▶), Kirkpatrick *et al.* (1983 ▶), Murty (1983 ▶), Pedersen (2002 ▶), Pèrez *et al.* (2001 ▶), Sinibaldi *et al.* (2007 ▶) and Spinozzi *et al.* (2007 ▶, 2010 ▶), as detailed in the supporting information.
