# Supplementary material for: GENFIT: software for the analysis of small-angle X-ray and neutron scattering data of macro­molecules in solution
Source: J Appl Crystallogr. 2014 May 10;47(Pt 3):1132–9. doi: 10.1107/S1600576714005147 (PMC4038801; doi:10.1107/S1600576714005147)
Supplement: Supplementary file 1 [file j-47-01132-sup1.pdf]

GENFIT: software for the analysis of small-angle X-ray and neutron scattering data of macromolecules in solution

F. Spinozzi,[a]* C. Ferrero,[b] M. G. Ortore,[a] A. De Maria Antolinos[b] and P. Mariani[a]

[a] *Department DiSVA, Marche Polytechnic University and CNISM, Via Brecce Bianche, I-60131 Ancona, Italy, and* [b] *European Synchrotron Radiation Facility, Grenoble, France. E-mail: f.spinozzi@univpm.it*

Supplementary Material

S1. List of models

The list of main models available in the current version of GENFIT is reported hereafter. Each model is briefly described and labeled with its number μ, enclosed in brackets, reflecting the historical order of the models integrated into GENFIT. There are other models in GENFIT that are not listed hereafter. Actually, on the one hand these models are very complex, however on the other hand they are very specific. We have decided to omit them in order to distribute a software tool of general use. The authors of GENFIT are willing to distribute a version of the software that includes all models upon request.

S1.1. Asymptotic behaviours

Guinier's law (16)

$$I_{16}(q) \;=\; \exp\left(-\frac{q^2 R_g^2}{3}\right) \quad \text{with } qR_g \leq C \tag{S1}$$

The two parameters of the model are the gyration radius, R_g, and the validity upper limit of the law, i.e. C. The user should define C as a fixed parameter (`Flag=0`). Typical C values range between 1 and 2 (Glatter & Kratky, 1982; Pèrez *et al.*, 2001).

Porod's law (22)

$$I_{22}(q) \;=\; \frac{2\pi S}{q^4 V^2} \tag{S2}$$

The two parameters of the model are the particle surface S and the volume V (Glatter & Kratky, 1982).

Debye's law (17)

$$I_{17}(q) \;=\; 2\frac{\exp(-R_g^2 q^2) + R_g^2 q^2 - 1}{R_g^4 q^4} \quad \text{with } qR_g \leq C \tag{S3}$$

Eq. S3 represents the form factor of a Gaussian chain (model (1), see Sect. S1.3), which is used to reproduce the asymptotic behaviour of a scattering pattern at low q to calculate the radius of gyration, R_g of a disordered chain. Hence, the two model parameters are R_g and the validity upper limit C of the approximation. The user should define C as a fixed parameter (`Flag=0`). Typical values of C are around 3 (Glatter & Kratky, 1982; Pèrez *et al.*, 2001).

Beaucage model of polymeric mass fractals (58) This model uses a combined Guinier/power law, able to describe multiple size-scale structures (Beaucage, 1996),

$$\begin{aligned} I_{58}(q) &= G\exp\left(-\frac{R_g^2q^2}{3}\right) + B\exp\left(-\frac{R_{sub}^2q^2}{3}\right)\left[\frac{((kqR_g/\sqrt{6}))^3}{q}\right]^P \\ &+G_s\exp\left(-\frac{R_s^2q^2}{3}\right) + B_s\left[\frac{((k_sqR_s/\sqrt{6}))^3}{q}\right]^{P_s} \end{aligned} \quad \text{(S4)}$$

The model parameters are described in the cited reference.

S1.2. Geometric solids

Homogeneous sphere (18)

$$I_{18}(q) = \left[\frac{4\pi}{3}R^3(\rho-\rho_0)\Phi(qR)\right]^2 \quad \text{(S5)}$$

$$\Phi(x) = 3\frac{\sin x - x\cos x}{x^3} \quad \text{(S6)}$$

The parameters are scattering length densities of the sphere and the matrix , ρ and ρ_0, respectively, and the sphere radius R.

Two-density level sphere (19)

$$\begin{aligned} I_{19}(q,R,\delta) &= \left\{\frac{4\pi}{3}\Big[(\rho_e-\rho_0)(R+\delta)^3\Phi\left(q(R+\delta)\right)\right. \\ &\left.+(\rho_i-\rho_e)R^3\Phi(qR)\Big]\right\}^2 \end{aligned} \quad \text{(S7)}$$

The model parameters are scattering length densities of the inner sphere, the outer sphere and the matrix, ρ_i, ρ_e and ρ_0, respectively, the inner sphere radius, R, and the shell thickness δ.

Three-density level sphere (42) This model is a straightforward extension of model (19) (Glatter & Kratky, 1982).

Two-density level cylinder (6)

$$\begin{aligned} I_6(q) &= \int_0^{\pi/2} d\beta \sin\beta \, [A_6(q)]^2 && \text{(S8)} \\ A_6(q) &= 2\pi L \frac{\sin\left(\frac{1}{2}qL\cos\beta\right)}{\frac{1}{2}qL\cos\beta} \\ &\times \left[(\rho_s - \rho_0)(R+\delta)^2 \frac{J_1(q(R+\delta)\sin\beta)}{q(R+\delta)\sin\beta}\right. \\ &\times \left. +(\rho_c - \rho_s)R^2 \frac{J_1(qR\sin\beta)}{qR\sin\beta}\right] && \text{(S9)} \end{aligned}$$

where $J_1(x)$ is the 1st order Bessel function of the first kind. Model parameters are: the inner cylinder radius R; the external shell thickness δ; the cylinder height L; the scattering length densities of shell, matrix and core, ρ_s, ρ_0 and ρ_c, respectively. Notice that in this model the user can also declare the cylinder volume V as a free parameter: in this case one of the other three geometric parameters L, R or δ should be defined as "constrained parameter", by using `Flag=3`.

Hollow cylinder with two coatings of different scattering length density (7)

$$\begin{aligned} I_7(q) &= \int_0^{\pi/2} d\beta \sin\beta \left\{ 2\pi L \frac{\sin\left(\frac{1}{2}qL\cos\beta\right)}{\frac{1}{2}qL\cos\beta} \right. \\ &\times \left. \sum_{k=1}^{4} (\rho_k - \rho_{k-1}) R_k^2 \frac{J_1(q(R_k \sin\beta)}{qR_k \sin\beta} \right\}^2 && \text{(S10)} \end{aligned}$$

The model parameters are: the cylinder height L; the radius of the cylindrical hole R_h; the internal, the core and the external shell thicknesses δ_{in}, δ_{core}, δ_{out}, respectively; the scattering length densities of external, core and internal shell ρ_k (corresponding to $k = 1, 2, 3$); the matrix scattering length density $\rho_0 = \rho_4$. The summation is over the four radii R_k defined as $R_1 = R_h + \delta_{in} + \delta_{core} + \delta_{out}$, $R_2 = R_h + \delta_{in} + \delta_{core}$, $R_3 = R_h + \delta_{in}$, $R_4 = R_h$.

Two-density-level three-axial ellipsoid (23)

$$
\begin{aligned}
I_{23}(q) &= \frac{2}{\pi}\int_0^{\pi/2}\int_0^{\pi/2}\sin\beta\, d\beta\, d\alpha\,[A_{23}(q)]^2 && \text{(S11)}\\
A_{23}(q) &= \frac{4\pi}{3}\sum_{k=0}^{1}(A+k\delta)(B+k\delta)(C+k\delta)(\rho_{k+1}-\rho_k)\\
&\times\Phi(q\{[(A+k\delta)^2\sin^2\alpha+(B+k\delta)^2\cos^2\alpha]\sin^2\beta\\
&+(C+k\delta)^2\cos^2\beta\}^{1/2}) && \text{(S12)}
\end{aligned}
$$

The model parameters are: the semiaxes of the inner ellipsoid, A, B and C; the shell thickness δ; the scattering length densities of matrix, ellipsoidal shell and inner ellipsoid, ρ_0, ρ_1 and ρ_2, respectively.

Two-density-level three-axial ellipsoid and DLVO potential under RMSA approximation (29) The form factor is the same as in model (23). The structure factor $S(q)$ is calculated in the framework of the rescaled mean spherical approximation (RMSA) with the Derjaguin-Landau-Verwey-Overbeek (DLVO) interaction potential. Details can be found in (Hayter & Penfold, 1981) and (Hansen & Hayter, 1982). The scattering intensity is the product of the form factor and the so-called "measured" (or "effective") structure factor $S_M(q)$ according to

$$
\begin{aligned}
I_{29}(q) &= I_{23}(q)S_M(q) && \text{(S13)}\\
S_M(q) &= 1+\frac{[I'_{23}(q)]^2}{I_{23}(q)}[S(q)-1] && \text{(S14)}\\
I'_{23}(q) &= \frac{2}{\pi}\int_0^{\pi/2}\int_0^{\pi/2}\sin\beta\, d\beta\, d\alpha\, A_{23}(q) && \text{(S15)}
\end{aligned}
$$

Two-density level cylinder and DLVO potential under RPA approximation (56) In this model the form factor reported in Eq. S9 is combined with the structure factor calculated using the DLVO potential within the random phase approximation (RPA). Details of the model can be found in (Ortore *et al.*, 2009). The scattering intensity is

expressed as:

$$I_{56}(q) = I_6(q)S_M(q) \tag{S16}$$

$$S_M(q) = 1 + \frac{[I'_6(q)]^2}{I_6(q)}[S(q) - 1] \tag{S17}$$

$$I'_6(q) = \int_0^{\pi/2} d\beta \sin\beta \, A_6(q) \tag{S18}$$

Two-density-level sphere and DLVO potential under RMSA approximation (59) The form factor is the same as in the model (19). The "measured" structure factor $S_M(q)$ is same as in the model (29) (see Eq. S14).

Two-density level tri-axial ellipsoid and DLVO potential under RPA approximation (61) The form factor is the same as in the model (23). The structure factor is calculated using the DLVO potential within the RPA.

Two-density level spherocylinder and DLVO potential under RPA approximation (63) The form factor of the spherocylinder is

$$I_{63}(q) = P_{63}(q)S_M(q) \tag{S19}$$

$$S_M(q) = 1 + \frac{[P'_{63}(q)]^2}{P_{63}(q)}[S(q) - 1] \tag{S20}$$

$$P_{63}(q) = \int_0^{\pi/2} d\beta \sin\beta \, [A_{63}(q)]^2 \tag{S21}$$

$$P'_{63}(q) = \int_0^{\pi/2} d\beta \sin\beta \, A_{63}(q) \tag{S22}$$

$$\begin{aligned} A_{63}(q) = {} & 4\pi \sum_{k=1}^{2} (\rho_k - \rho_{k+1}) R_k^2 \\ & \times \int_0^1 X[R + R_k(1 - X^2)^{1/2}] J_0(qR_k X \sin\beta) \\ & \times \frac{\sin[q(R + R_k(1 - X^2)^{1/2}) \cos\beta]}{q\left(R + R_k(1 - X^2)^{1/2}\right) \cos\beta} \, dX \end{aligned} \tag{S23}$$

where $J_0(x)$ is the 0th order Bessel function of the first kind, $R = L/2$, L is the length of the cylinder, R_1 is the inner cylinder radius and the inner radius of the two hemispherical caps, $R_2 = R_1 + \delta$, δ is the shell thickness. The scattering length densities of core, shell and matrix are ρ_1, ρ_2 and ρ_3, respectively. The structure factor $S(q)$ is calculated using the DLVO potential within the random phase approximation (RPA).

S1.3. Disordered chains

Gaussian chain (1) The form factor is described by Debye's law (Eq. S3). The model is applied to the full experimental q-range. The only free parameter is R_g.

Gaussian chain with finite cross section (2) This model is obtained by multiplying the form factor of the model (1) by the cylindrical cross section,

$$S_{sc}(q) = \left[2\frac{J_1(Rq)}{Rq}\right]^2 \tag{S24}$$

Parameters of the model are R_g and the radius of the chain cross section, R.

Worm-like model without excluded volume effect (3) This model was developed by (Pedersen & Schurtenberger, 1996). The reader is referred to the original article for details. Model parameters are: the statistical segment (Kuhn) length b, and the contour length, L.

Worm-like model without excluded volume effect and finite cross-section (4) This model is obtained by multiplying the form factor of the previous model (3) by the cylindrical cross section (Eq. S24).

Worm-like model without excluded volume effect and finite two-density level cross-section (10) This model results from the product of the form factor of model (3) times a cylindrical two-density level cross section:

$$S_{sc,2}(q) \quad = \quad 4\pi^2 \left[(\rho_s - \rho_0)(R+\delta)^2 \frac{J_1(q(R+\delta))}{q(R+\delta)} + (\rho_c - \rho_s) R^2 \frac{J_1(qR)}{qR} \right]^2 \quad \text{(S25)}$$

The symbols in Eq. S25 have the same meanings as in the previous formulas.

Worm-like model with excluded volume effect and finite cross-section (11) This is like model (4) but allows for the effect of excluded volume, as described in (Pedersen & Schurtenberger, 1996). The related Fortran subroutines were kindly made available by J. Skov Pedersen.

Worm-like model with excluded volume effect and finite two-density level cross-section (12) This is similar to model (11), but using the cross section of Eq. S25.

Sphere with attached gaussian chains (5) This model (by J. Skov Pedersen) is reported in (Pedersen, 2002).

Worm-like model with excluded volume effect and finite two-density level cross-section and DLVO potential under RPA approximation (47) In this model the form factor of model (12) is combined with the structure factor of model (58). See (Barbosa *et al.*, 2010) for further details.

S1.4. PDB structures

Monte Carlo form factor of a PDB structure with a Gaussian hydration shell (9) The shape of the particle, considered homogeneous, is evaluated by the envelope of all the van der Waals spheres centrered on the atomic coordinates. The Monte Carlo

method is used to determine the distance distribution histogram. A Gaussian transition layer is introduced to smooth out the particle border. Details are reported in (Cinelli *et al.*, 2001) and (Spinozzi *et al.*, 2002). Model parameters are the ordinal number of the PDB file and the standard deviation of the Gaussian transition layer, σ. "Preliminary parameters" of the model are: `Random Points Number` (used in the Monte Carlo sampling) and `Radial grid amplitude` (used to calculate the distance distribution histograms). Distance distribution histograms are saved in the file `gen<code><pp>pdb.pr` (see Sect. S4) and can be re-used in other GENFIT runs.

Monte Carlo form factor of a PDB structure with a solvation shell of different scattering length density (13) This is akin to model (9). The shape of the solvation shell around the protein is calculated by adding a constant thickness δ to the envelope function. Three pair distance distribution histograms $p_{ij}(r)$, corresponding to core-core ($p_{cc}(r)$), shell-shell ($p_{ss}(r)$) and core-shell ($p_{cs}(r)$) terms and two single distance distribution histograms $p'_i(r)$ corresponding to centre-core ($p'_c(r)$) and centre-shell ($p'_s(r)$) are evaluated by a Monte Carlo sampling method. Shell and core volumes, V_s and V_c estimated through the Monte Carlo method, can be isotropically expanded or contracted. The details of the model are described in (Sinibaldi *et al.*, 2007) and (Spinozzi *et al.*, 2007). Model parameters are: the ordinal number of the PDB file; the thickness o δ f the shell; core, shell and matrix scattering length densities, ρ_c, ρ_s and ρ_0, respectively; core and shell volumes, V_c and V_s. "Preliminary parameters" of the model are: `Random Points Number` (used in the Monte Carlo sampling) and `Radial grid amplitude` (used to calculate the distance distribution histograms). Distance distribution histograms are saved in the file `gen<code><pp>pdb.pr` and can be re-used for

other GENFIT runs. The scattering intensity is calculated according to

$$
\begin{aligned}
I_{13}(q) &= (\rho_c - \rho_0)^2 V_c^2 P_{cc}(q) + (\rho_s - \rho_0)^2 V_s^2 P_{ss}(q) \\
&\quad +2(\rho_c - \rho_0)(\rho_s - \rho_0) V_c V_s P_{cs}(q) \qquad \text{(S26)}
\end{aligned}
$$

where $P_{ij}(q)$ are the isotropic Fourier transforms of the corresponding pair distribution histograms,

$$
P_{ij}(q) = \int_0^\infty p_{ij}(r) \frac{\sin(qr)}{qr} dr \qquad \text{(S27)}
$$

Monte Carlo form factor of a PDB structure with a solvation shell of different scattering length density and DLVO potential under RPA approximation (28) The form factor of interacting proteins is calculated as in model (13). The structure factor is built with the DLVO potential in the frame of RPA. Distance distribution histograms are saved in the file `gen<code><pp>pdb.pr` and can be re-used in other runs of GENFIT. The scattering intensity is calculated as

$$
\begin{aligned}
I_{28}(q) &= I_{13}(q)\, S_M(q) \qquad \text{(S28)} \\
S_M(q) &= 1 + \frac{[I'_{13}(q)]^2}{I_{13}(q)} [S(q) - 1] \qquad \text{(S29)} \\
I'_{13}(q) &= (\rho_c - \rho_0) V_c P'_c(q) + (\rho_s - \rho_0) V_s P'_s(q) \qquad \text{(S30)}
\end{aligned}
$$

where $P'_i(q)$ are the isotropic Fourier transforms of the corresponding single distribution histograms,

$$
P'_i(q) = \int_0^\infty p'_i(r) \frac{\sin(qr)}{qr} dr \qquad \text{(S31)}
$$

All-atoms form factor of a PDB structure with solvation shell of dummy atoms and multipole expansion average (15) A full description of the method is given in Ref. (Ortore *et al.*, 2009). The contribution of each atom present in the PDB file is taken into

account through its atomic structure factor. The displaced solvent contribution is calculated considering Gaussian dummy spheres centrered on the PDB atomic positions. Solvent molecules in contact with the macromolecule are also described by dummy Gaussian spheres: their number and their geometrical coordinates are found by burying the macromolecule in a tetrahedrally close packed assembly of dummy spheres. Fourier transforms of partial amplitudes are saved in the file gen<code><pp>pdb.pr and can be re-used for other GENFIT computational tasks.

Interacting PDB structures: all-atoms form factor of a PDB structure with solvation shell of dummy atoms and multipole expansion average and first order density expansion of $u_{ij}(r)$ (25) The form factor of interacting proteins is calculated according to model (15). The Ashcroft-Langreth partial structure factors are obtained by a first-order power series expansion of the protein-protein correlation functions, $g_{ij}(r)$ in terms of the total particles' concentration. All details of the method are described in (Spinozzi *et al.*, 2002). The Fourier transforms of the partial amplitudes are saved in the file gen<code><pp>pdb.pr and can be re-used for other runs of GENFIT.

Interacting PDB structures: all-atoms form factor of a PDB structure with solvation shell of dummy atoms, multipole expansion average and DLVO potential under RPA approximation (46) The form factor of interacting proteins is calculated according to model (15). The "measured" structure factor is computed using the DLVO potential in the RPA framework and combined into the form factor by an equation similar to Eq. S29. The Fourier transforms of the partial amplitudes are saved in the file gen<code><pp>pdb.pr and can be re-used in other runs of GENFIT.

S1.5. Self-assembled amphiphilic systems

These models have been developed to analyse SAS data of amphiphilic molecules, such as lipids or detergents.

Multilamellar vesicle (24) The amphiphilic spherical layer is simulated by three domains, each of constant scattering length density. Parameters characterising this model are: the inner radius of the vesicle R_0; the thicknesses of polar head, alkyl chains and terminal groups R_1, R_2 and R_3, respectively; the corresponding scattering length densities ρ_1, ρ_2 and ρ_3; the thickness of the water layer between two amphiphilic bilayers R_w; the scattering length density ρ_0 of water; the number n of bilayers. The form factor expression is:

$$\begin{aligned} I_{24}(q) &= \frac{4}{3}\pi \sum_{i=1}^{n} (\rho_0 - \rho_1)\{[R_0 + (i-1)A]^3 \Phi(q[R_0 + (i-1)A]) \\ &\quad -[R_0 - R_w + iA]^3 \Phi(q[R_0 - R_w + iA])\} \\ &\quad +(\rho_1 - \rho_2)\{[R_0 + R_1 + (i-1)A]^3 \Phi(q[R_0 + R_1 + (i-1)A]) \\ &\quad -[R_0 - R_w - R_1 + iA]^3 \Phi(q[R_0 - R_w - R_1 + iA])\} \\ &\quad +(\rho_2 - \rho_3)\{[R_0 + R_1 + R_2 + (i-1)A]^3 \Phi(q[R_0 + R_1 + R_2 + (i-1)A]) \\ &\quad -[R_0 - R_w - R_1 - R_2 + iA]^3 \Phi(q[R_0 - R_w - R_1 - R_2 + iA])\} \end{aligned} \quad \text{(S32)}$$

where $A = R_w + 2(R_1 + R_2 + R_3)$.

Multilamellar vesicle with smoothed scattering length density profile (34) Akin to model (24). The scattering length density profile is modelled by a four-level function (corresponding to solvent, headgroup, alkyl chain and terminal group domains) with transitions between levels described by the error function (z) (Spinozzi *et al.*, 2010),

$$\rho(z) = \rho_0 + \frac{1}{2}\sum_{i=1}^{3}(\rho_{i-1} - \rho_i)\left[\left(\frac{z - z_i}{2^{1/2}\sigma_i}\right) - \left(\frac{z + z_i}{2^{1/2}\sigma_i}\right)\right] \quad \text{(S33)}$$

where z_i and σ_i represent position and standard deviation of the i-th step of the error function, respectively, and $z_3 = R_3$, $z_2 = R_3 + R_2$, $z_1 = R_3 + R_2 + R_1$. For the other symbols, see above. The scattering intensity reads

$$I_{34}(q) = [A_{34}(q)]^2 \tag{S34}$$

$$A_{34}(q) = \sum_{j=1}^{n} V(q, R_0 + (j-1)(R_w + 2z_1)) \tag{S35}$$

$$V(q,R) = \frac{4}{3}\pi \sum_{i=1}^{3} (\rho_i - \rho_{i-1}) e^{-(q\sigma_i)^2/2} \times\{(R+z_i)^3 \Phi(q(R+z_i)) - (R-z_i)^3 \Phi(q(R-z_i)) \tag{S36}$$

$$+3\sigma_i^2 [(R+z_i) j_0(q(R+z_i)) - (R-z_i) j_0(q(R-z_i))]\} \tag{S37}$$

where $j_0(x)$ is the 0th Bessel functions of fractional order.

Infinite multilamella with smoothed scattering length density profile and MCT theory (35) Each multilamella is formed by $M \geq 1$ flat, infinitely extended bilayers, the scattering length density profile of each bilayer being described by the smooth function in Eq. S33. The structure factor of a stack of parallel multilamellae is described by the modified Caillé theory (MCT), which exhibits three varying parameters: the mean number of coherently scattering multilamellae, N; the repeat distance c and the Caillé parameter η_1 to quantify stack fluctuations. For details see Ref. (Zhang *et al.*, 1994) and Ref. (Frühwirth *et al.*, 2004). The scattering intensity is

$$I_{35}(q) = \frac{2\pi}{q^2} |A_{35}(q)|^2 S_{MCT}(q) \tag{S38}$$

$$A_{35}(q) = \sum_{j=1}^{M} e^{\imath q(j-1)(R_w+2z_1)} F(q) \tag{S39}$$

$$F(q) = 2\sum_{i=1}^{3} z_i (\rho_i - \rho_{i-1}) j_0(qz_i) e^{-(q\sigma_i)^2/2} \tag{S40}$$

$$S_{MCT}(q) = 1 + \frac{2}{N} \sum_{m=1}^{N-1} (N-m)\cos(mqc) \times(\pi m)^{-(c/(2\pi))^2 q^2 \eta_1} e^{-\gamma (c/(2\pi))^2 q^2 \eta_1} \tag{S41}$$

where γ is Euler's constant.

Bicelle model with two smoothed scattering length density profiles and 2D and 1D finite paracrystal (48) A bicelle is a flat bilayer in the form of a disk with radius R surrounded by a rim, modeled as the external part of a torus with major radius R. The two scattering length density profiles, one in the direction perpendicular to the flat bilayer and the other in the radial direction of the circular section of the torus, are described by Eq. S33. Bicelles can be correlated in the direction perpendicular to their plane (stacking interaction) or in their plane, assuming a two-dimensional hexagonal lattice. The two correlations are described by the structure factor of a finite paracrystal (Matsuoka *et al.*, 1987), in one or two dimensions. The number N_c of vertically interacting bicelles, their repeat distance, c, and the perpendicular distortion parameter, g_c are the parameters of the one-dimensional structure factor. For the horizontal order, the parameters are: the number N_a of bicelles in one direction of the hexagonal lattice; the lattice parameter a; the parallel distortion parameter, g_a. The resulting scattering intensity writes

$$I_{48}(q) = \frac{2}{\pi}\int_0^{\pi/2} \sin\beta_q |A(q,\beta_q)|^2 \int_0^{\pi/2} d\alpha_q S_{PT}(\mathbf{q}) \quad \text{(S42)}$$

$$\begin{aligned} A(q,\beta_q) &= 2\pi R^2 \frac{J_1(qR\sin\beta_q)}{qR\sin\beta_q} F(q\cos\beta_q) - 4\pi \int_0^{\infty} u^2\, du\, \frac{d\rho_e(u)}{du} \\ &\times \int_0^{\pi/2} d\beta \sin^2\beta\, j_0(qu\sin\beta\cos\beta_q)(R+u\cos\beta) \\ &\times J_0(q[R+u\cos\beta]\sin\beta_q) \end{aligned} \quad \text{(S43)}$$

$F(q)$ and $\frac{d\rho_e(u)}{du}$ are the scattering amplitude of the flat part of the bicelle and the first derivative of the scattering length density in the radial direction u of the circular

section of the torus:

$$F(q) = 2\sum_{i=1}^{3} z_i(\rho_{i,f} - \rho_{i-1,f})e^{-(q\sigma_{i,f})^2/2}\frac{\sin(qz_{i,f})}{qz_{i,f}} \quad \text{(S44)}$$

$$\frac{d\rho_e(z)}{dz} = (2\pi)^{-1/2}\sum_{i=1}^{3}(\rho_{i-1,e} - \rho_{i,e})\frac{1}{\sigma_{i,e}} \times \left[e^{-\{(z-z_{i,e})/\sigma_{i,e}\}^2/2} - e^{-\{(z+z_{i,e})/\sigma_{i,e}\}^2/2}\right] \quad \text{(S45)}$$

(S46)

For the flat ($k = f$) and the rim ($k = e$) region of the bicelle $z_{i,k}$ and $\sigma_{i,k}$ stand for position and standard deviation, respectively, of the i-th step of the error function and $z_{3,k} = R_{3,k}$, $z_{2,k} = R_{3,k} + R_{2,k}$, $z_{1,k} = R_{3,k} + R_{2,k} + R_{1,k}$. $R_{1,k}$, $R_{2,k}$ and $R_{3,k}$ are the thicknesses of polar head, alkyl chains and terminal groups, respectively. The corresponding scattering length densities are $\rho_{1,k}$, $\rho_{2,k}$ and $\rho_{3,k}$. The scattering length density of water is $\rho_{0,k} \equiv \rho_0$. $S_{PT}(\mathbf{q})$ is the paracrystal structure factor:

$$S_{PT}(\mathbf{q}) = \prod_{k=1}^{3} \mathrm{Re}\left[\frac{1+F_k}{1-F_k} - \frac{2F_k(1-F_k^{N_k})}{N_k(1-F_k)^2}\right] \quad \text{(S47)}$$

$$F_k = e^{-(\mathbf{q}\cdot\boldsymbol{\Delta}_k)^2/2}e^{i\mathbf{q}\cdot\mathbf{a}_k} \quad \text{(S48)}$$

where: $N_1 = N_2 = N_a$ and $N_3 = N_c$. $\mathbf{q} = (q\sin\beta_q\cos\alpha_q, q\sin\beta_q\sin\alpha_q, q\cos\beta_q)$ is the scattering vector. $\mathbf{a}_1 = (a,0,0)$, $\mathbf{a}_2 = (a/2, a\sqrt{3}/2, 0)$ and $\mathbf{a}_3 = (0,0,c)$ are the unit cell vectors of the hexagonal Bravais lattice. $\boldsymbol{\Delta}_1 = \boldsymbol{\Delta}_2 = a(g_a, g_a, 0)$ and $\boldsymbol{\Delta}_3 = c(0,0,g_c)$ are the vectors of the semiaxes of the lattice distorsion ellipsoids.

S2. List of polydispersity models

In the following, the expressions and the parameters of the seven polydispersity models are reported.

1. Normalized Gaussian distribution (`Kind=1`),

$$f_1(X) = \left[(2\pi)^{1/2} < X > \xi\right]^{-1} \exp\left\{-\frac{[X- < X >]^2}{2 < X >^2 \xi^2}\right\} \quad \text{(S49)}$$

where the two parameters $< X >$ and $\xi \equiv [(< X^2 > - < X >^2)/ < X >^2]^{1/2}$ are the average value of X and its dispersion, respectively. The normalization condition reads

$$\int_{-\infty}^{\infty} dX f_1(X) \quad = \quad 1. \tag{S50}$$

2. Normalized log-normal distribution (`Kind=2`),

$$\begin{aligned} f_2(X) &= X^{-1}\left[2\pi \log(1+\xi^2)\right]^{-1/2} \\ &\times \exp\left\{-\frac{\log^2\left[X < X >^{-1}(1+\xi^2)^{1/2}\right]}{2\log(1+\xi^2)}\right\}. \end{aligned} \tag{S51}$$

The normalization condition is

$$\int_{0}^{\infty} dX f_2(X) \quad = \quad 1. \tag{S52}$$

3. Normalized Lorentzian distribution (`Kind=3`),

$$f_3(X) \quad = \quad [\pi < X > \xi]^{-1}\left[1+\frac{[X - < X >]^2}{< X >^2 \xi^2}\right]^{-1}. \tag{S53}$$

The normalization condition is

$$\int_{-\infty}^{\infty} dX f_3(X) \quad = \quad 1. \tag{S54}$$

4. Normalized double log-normal distribution (`Kind=4`).

$$\begin{aligned} f_4(X) &= \alpha X^{-1}\left[2\pi \log(1+\xi_1^2)\right]^{-1/2} \exp\left\{-\frac{\log^2\left[X < X >_1^{-1}(1+\xi_1^2)^{1/2}\right]}{2\log(1+\xi_1^2)}\right\} \\ &+(1-\alpha)X^{-1}\left[2\pi \log(1+\xi_2^2)\right]^{-1/2} \\ &\times \exp\left\{-\frac{\log^2\left[X < X >_2^{-1}(1+\xi_2^2)^{1/2}\right]}{2\log(1+\xi_2^2)}\right\}, \end{aligned} \tag{S55}$$

with five parameters: two averages $< X >_1$ and $< X >_2$, two associated dispersions, ξ_1 and ξ_2 and the relative weight α, comprised between 0 and 1. The normalization condition is

$$\int_{0}^{\infty} dX f_4(X) \quad = \quad 1. \tag{S56}$$

5. Eight cubic B-splines normalized distribution (`Kind=5`),

$$f_5(X) \;=\; \frac{11}{X_{c,m,k,\max}\sum_{n=1}^{8} c_n}\sum_{n=1}^{8} c_n B_{3,n}(X), \qquad \text{(S57)}$$

where $B_{3,n}(X)$ are bell-shaped third degree polynomials defined in the range $[0, X_{c,m,k,\max}]$, $X_{c,m,k,\max}$ being both the value at which the distribution function becomes zero and the first of the nine parameters that characterize the distribution, together with the eight unknown weights c_n. Notice the normalization condition:

$$\int_0^{X_{c,m,k,\max}} dX f_5(X) \;=\; 1. \qquad \text{(S58)}$$

6. Ten cubic B-splines normalized distribution over a logarithmic scale (`Kind=6`),

$$f_6(X) \;=\; \frac{13}{(X_{c,m,k,\mathrm{up}} - X_{c,m,k,\mathrm{low}})\sum_{n=1}^{10} c_n}\sum_{n=1}^{10} c_n B_{3,n}(\log X), \qquad \text{(S59)}$$

where $B_{3,n}(\log X)$ are defined in the fixed interval $[X_{c,m,k,\mathrm{low}}, X_{c,m,k,\mathrm{up}}]$ so that the model parameters are the ten weights c_n. The normalization condition is:

$$\int_{X_{c,m,k,\mathrm{low}}}^{X_{c,m,k,\mathrm{up}}} dX f_6(X) \;=\; 1. \qquad \text{(S60)}$$

7. Eight cubic B-splines normalized distribution over a logarithmic scale (`Kind=7`),

$$f_7(X) \;=\; \frac{11}{(X_{c,m,k,\max} - X_{c,m,k,\min})\sum_{n=1}^{8} c_n}\sum_{n=1}^{8} c_n B_{3,n}(\log X), \qquad \text{(S61)}$$

where $B_{3,n}(\log X)$ are defined in the interval $[X_{c,m,k,\min}, X_{c,m,k,\max}]$, with both limits taken as model parameters together with the eight weights c_n. The normalization condition is:

$$\int_{X_{c,m,k,\min}}^{X_{c,m,k,\max}} dX f_7(X) \;=\; 1. \qquad \text{(S62)}$$

S3. Minimisation methods

(i) Monkey is a simple-minded method, where each parameter is randomly moved within its validity range; the final set of parameters is the one that, after the selected maximum number of iterations, has provided the minimum value of χ^2.

(ii) The simulated annealing method is particularly suitable when several local minima of the functional coexist. Parameters of the method are the starting and final values of the generalized temperature (T_i^* and T_f^*, respectively) as well as the number of sub-runs (N_s) and the number of cycles (N_c) per sub-run (Kirkpatrick *et al.*, 1983). The starting generalized temperature is typically set to $T_i^* = \chi_0^2/2$, being χ_0^2 the value of χ^2 corresponding to the initial guess of the parameters. T^* is decreased in N_s steps down to T_f^* (which should be as large as the presumed final χ^2 value), according to a geometric series behaviour. Typical values of N_s and N_c are comprised between 10 and 50.

(iii) The simplex method is based on a popular algorithm for solving numerically linear programming problems (Murty, 1983). The user is requested to enter the maximum number of iterations.

(iv) The quasi-Newton method is implemented using the `zxmin` subroutine from the IMSL library (Aird, 1984). The only parameter that the user is requested to enter is the maximum number of iterations. Since in this method the Hessian matrix is evaluated, GENFIT calculates the correlation matrix among the fitting parameters and report it in the file `gen<code>.out`.

The minimisation methods are applied by GENFIT in the order from (i) to (iv). These methods that have not been selected by the user are skipped.

S4. List of GENFIT input and output files

gen<code>.dat: the input file of the program, created and uploaded by the GUI.

gen<code>.out: the main output of the program. It is an ASCII file in which all the results of the optimisation, including fitting parameters, are reported.

gen<code>.par: the ASCII file of the fitting parameters. It can be used to define the validity ranges of f-type parameters, as described in Sec. 2.9.

gen<code>.log: an ASCII file that reports the final values of all parameters (κ_c, B_c, $w_{c,m}$, $X_{c,m,k}$ together with p- and f-type parameters, written as a list of statements of the type <varname>=value and e_<varname>=value, where <varname> is the internal name of the variable used by GENFIT in the calculation and e_<varname> is the name of the corresponding standard deviation. This file can be easily included in a script.

gen<code><nn>.fit: a six-column ASCII file that contains the c-curve best fit. <nn> is the two-digit numeric code of the c-curve. In the columns 1-6 the following values are written: q, $I_{\exp,c}(q)$, $\log[I_{\exp,c}(q)]$, $\hat{I}_c(q)$, $\log[\hat{I}_c(q)]$, $\sigma_c(q)$ and $S_{M,c}(q)$ (see Sec. S1 for the meaning of the last value).

gen<code><nn>.pq: a multi-column ASCII file comprising all the calculated curves (Eq. 2). Curves are calculated over the q-range $[0, 100\pi/R_{\max}]$, $R_{\max}$ being the anticipated maximum value of the intra-particle distance, introduced as input parameter by the user. The columns are formed by the following values: q, $I_c(q)$, $I_{c,1}(q)$, $w_{c,1}I_{c,1}(q)$, $I_{c,2}(q)$, $w_{c,2}I_{c,2}(q)$, $\ldots$ $I_{c,M_c}(q)$, $w_{c,M_c}I_{c,M_c}(q)$.

gen<code><nn>.abs: an ASCII file with the three columns: q, $\hat{I}_c(q)$, 1.0. This file can be used as an experimental data file for other GENFIT calculations.

gen<code><nn>.sim: a three-column ASCII file, where the columns are: q, $\hat{I}_c(q)$ randomly moved within its error bar, $k[\hat{I}_c(q)]^\alpha$. This file can be used as an experimental data file for other calculations with GENFIT.

IUCr macros version 2.1.5: 2012/03/07

gen<code><nn>.pr: a two-column ASCII file reporting the normalized isotropic Fourier transform of the fitting curve $I_c(q)$,

$$p_c(r) \quad = \quad \frac{2r}{\pi}\int_0^{100\pi/R_{\max}} dq\,\frac{I_c(q)}{I_c(0)} q\,\sin qr. \tag{S63}$$

gen<code>fit.gnu: a script to plot the best fit results of all the N_c SAS curves and the related Fourier transforms $p_c(r)$.

gen<code>pq.gnu: a script to plot the best fit results reported in the files gen<code><nn>.pq.

gen<code><pp>pdb.pr: an ASCII file that reports the Fourier transforms of the partial amplitudes, calculated for the models making use of the pp-th PDB file.

gen<code><nn><mm><varname>.dis: a multicolumn ASCII file including the polydispersity function $f_{c,m,k}(X_{c,m,k})$ (Eq. 3) of the parameter named <varname> by GENFIT and used in the m-th model of the c-curve (tagged with the two-digit codes <mm> and <nn>, respectively). The function is calculated for the values of $X_{c,m,k}$ used in the numerical integration carried out with the trapezoidal rule (see Sec. 2.7) and normalized to unit,

$$\hat{f}_{c,m,k}(X_{c,m,k}) \quad = \quad \frac{f_{c,m,k}(X_{c,m,k})}{\int_{X_{c,m,k,\mathrm{low}}}^{X_{c,m,k,\mathrm{up}}} dX_{c,m,k} f_{c,m,k}(X_{c,m,k})}. \tag{S64}$$

In the columns are arranged the following values: $X_{c,m,k}$, $10^{-8}\kappa_c\hat{f}_{c,m,k}(X_{c,m,k})$, $\hat{f}_{c,m,k}(X_{c,m,k})$, $10^{-8}\kappa_c\sigma[\hat{f}_{c,m,k}]$, $\sigma[\hat{f}_{c,m,k}]$, $f_{c,m,k}(X_{c,m,k})$,$\sigma[f_{c,m,k}]$, where $\sigma(x)$ indicates the standard deviation of x. Notice that 10^{-8} is the conversion factor from cm^{-1} to $\mathrm{Å}^{-1}$.

gen<code><nn><mm><varname>l.dis: a multicolumn ASCII file similar to the previous one, but calculated over a grid of 2^8 values in the range $[X_{c,m,k,\mathrm{low}}, X_{c,m,k,\mathrm{up}}]$.

gen<code><nn><mm>.ps: is a PostScript file generated by GENFIT for some models and featuring a representation of the particle obtained with the optimised parameters. The meaning of the two-digit codes <nn> and <mm> is the same as

above.

References

Aird, T. J. (1984). *The IMSL library, Sources and Development of Mathematical Software.* Englewood Cliffs, NJ, USA: Prentice-Hall Inc.

Barbosa, L. R., Ortore, M. G., Spinozzi, F., Mariani, P., Bernstorff, S. & Itri, R. (2010). *Biophys. J*, **98**, 147–157.

Beaucage, G. (1996). *J. Appl. Cryst.* **29**, 134–146.

Cinelli, S., Spinozzi, F., Itri, R., Carsughi, F., Onori, G. & Mariani, P. (2001). *Biophys. J.* **81**, 3522–3533.

Frühwirth, T., Fritz, G., Freiberger, N. & Glatter, O. (2004). *J. Appl. Cryst.* **37**, 703–710.

Glatter, O. & Kratky, O. (1982). *Small Angle X-ray Scattering.* Academic Press.

Hansen, J. P. & Hayter, J. B. (1982). *Mol. Phys.* **46**, 651–656.

Hayter, J. B. & Penfold, J. (1981). *Mol. Phys.* **42**, 109–118.

Kirkpatrick, S., Gelatt, C. D. & Vecchi, M. P. (1983). *Science*, **220**, 671–680.

Matsuoka, H., Tanaka, H., Hashimoto, T. & Ise, N. (1987). *Phys. Rev. B*, **36**(3), 1754–1765.

Murty, K. G. (1983). *Linear programming.* Wiley Subscription Services, Inc., A Wiley Company.

Ortore, M. G., Spinozzi, F., Mariani, P., Paciaroni, A., Barbosa, L. R. S., Amenitsch, H., Steinhart, M., Ollivier, J. & Russo, D. (2009). *J. R. Soc. Interface*, **6**, S619–S634.

Pedersen, J. S. (2002). In *Neutron, X-rays and Light. Scattering Methods Applied to Soft Condensed Matter*, edited by P. Lindner & T. Zemb, pp. 103–124. North-Holland.

Pedersen, J. S. & Schurtenberger, P. (1996). *Macromolecules*, **29**, 7602–7612.

Pèrez, J., Vachette, P., Russo, D., Desmadril, M. & Durand, D. (2001). *J. Mol. Biol.* **308**, 721–743.

Sinibaldi, R., Ortore, M. G., Spinozzi, F., Carsughi, F., Frielinghaus, H., Cinelli, S., Onori, G. & Mariani, P. (2007). *J. Chem. Phys.* **126**, 235101.

Spinozzi, F., Carsughi, F., Mariani, P., Saturni, L., Bernstorff, S., Cinelli, S. & Onori, G. (2007). *J. Phys. Chem. B*, **111**, 3822–3830.

Spinozzi, F., Gazzillo, D., Giacometti, A., Mariani, P. & Carsughi, F. (2002). *Biophys. J.* **82**, 2165–2175.

Spinozzi, F., Paccamiccio, L., Mariani, P. & Amaral, L. Q. (2010). *Langmuir*, **26**, 6484–6493.

Zhang, R., Suter, R. M. & Nagle, J. F. (1994). *Phys. Rev. E*, **50**, 5047–5060.
